# pH- and calcium-dependent solution-phase free fluoride in dental desensitizing gels: a preliminary in vitro study

**DOI:** 10.3389/fbioe.2026.1788855

**Published:** 2026-07-10

**Authors:** Paweł J. Piszko, Michał J. Kulus, Aleksandra Piszko, Jan Kiryk, Sylwia Kiryk, Julia Kensy, Agata Małyszek, Mateusz Michalak, Wojciech Dobrzyński, Jacek Matys, Maciej Dobrzyński

**Affiliations:** 1 Department of Biomedical Engineering, Faculty of Fundamental Problems of Technology, Wrocław University of Science and Technology, Wroclaw, Poland; 2 Division of Ultrastructural Research, Wroclaw Medical University, Wroclaw, Poland; 3 Department of Pediatric Dentistry and Preclinical Dentistry, Wroclaw Medical University, Wroclaw, Poland; 4 Dental Surgery Department, Wroclaw Medical University, Wroclaw, Poland; 5 Department of Biostructure and Animal Physiology, Wrocław University of Environmental and Life Sciences, Wroclaw, Poland; 6 Medical Center of Innovation, Wroclaw Medical University, Wroclaw, Poland; 7 Department of Dentofacial Orthopedics and Orthodontics, Division of Facial Abnormalities, Wroclaw Medical University, Wroclaw, Poland

**Keywords:** artificial saliva, calcium ions, dental gels, desensitizing gels, fluoride, *in vitro*

## Abstract

**Introduction:**

Dental hypersensitivity (DH) is a common clinical condition characterized by brief, intense pain. This condition is treated with fluoride-containing specialized dental gels. The effect of calcium ions present in saliva and pH of the oral cavity are indicated as factors affecting their clinical performance.

**Methods:**

In the presented study, two dental desensitizing gels were evaluated. Fourier Transform Infrared Spectroscopy (FTIR) spectra of the gels were registered and analyzed. Measurement of concentration of measurable free fluoride ions and pH *in vitro* assessment was performed using selective electrodes. Measurement with addition of TISAB witch CDTA was performed to evaluate the amount of fluoride bound within the gel matrix. Univariate and multifactorial ANOVA tests were used to analyze acquired results of measurable free fluoride in twelve distinct incubation solutions.

**Results and Discussion:**

The highest values of free, measurable fluoride were obtained after addition of TISAB with CDTA. However, in physiological study without ionic strength adjuster and chelator, the highest free, measurable fluoride observed in the artificial saliva solutions was 1,043 ppm for the SensiKIN desensitizing gel at pH 4.5 in the solution without calcium ion addition. The lowest value (658 ppm) was denoted for the Teeth Desensitiser TD gel at pH 7.0 with calcium ion addition. The presented amounts of fluoride pertain to measurable free fluoride in solution. It has been shown that the presence of calcium in saliva reduces the concentration of measurable free fluoride in solution (by as much as 137 ppm). Although the effect of presence of Ca^2+^ is pronounced, the initial pH remains the most significant variable. Observed levels of measurable free fluoride in solution might reflect initial and preliminary bioavailability under static *in vitro* conditions. Measurable free fluoride may serve as an indirect analytical proxy for potentially available fluoride under the tested *in vitro* conditions. However, it does not represent direct biological or clinical bioavailability. The evaluated desensitizing gels showed maximal free, measurable fluoride concentration under acidic conditions. For studies incorporating TISAB/CDTA, the most profound effect on F^−^ concentration was gel composition. Future formulation studies could investigate whether calcium- complexing or buffering components influence measurable solution-phase free fluoride under standardized *in vitro* conditions.

## 1 Introduction

DH is a prevalent clinical condition, manifested by a brief, intense pain that concerns exposed dentin as a consequence of thermal, chemical, or tactile stimulation. The condition is distinguished by the absence of any identifiable underlying etiology (Brännström, 1986; Davari et al., 2013). The prevalence of DH ranges from 10% to 30% of the adult population. However, more recent epidemiological data indicate even higher incidence rates among young adults and patients with periodontal disease (Bartold, 2006; Davari et al., 2013; Costa et al., 2014; Dionysopoulos et al., 2023). The predominant scientific consensus regarding the mechanism of DH development is encapsulated by the hydrodynamic theory. This theory posits that the application of stimuli to exposed dentinal tubules initiates fluid movement within the tubules, thereby stimulating mechanoreceptors in close proximity to the nerve endings of the pulp (Brännström, 1986). Recent studies have also highlighted the involvement of inflammatory mediators and ion channel regulation (TRPV1, ASIC3) in hypersensitive dentin. This extends the classical view of hydrodynamics to neurophysiological modulation (Zhang et al., 2023). It is evident that the primary objective of the majority of desensitizing agents is to restrict the movement of dentin fluid. This is achieved through the closure or narrowing of the dentinal tubules, or by reducing the excitability of pulpal nerves (Gillam et al., 1999; Porto et al., 2009).

**FIGURE 1:**
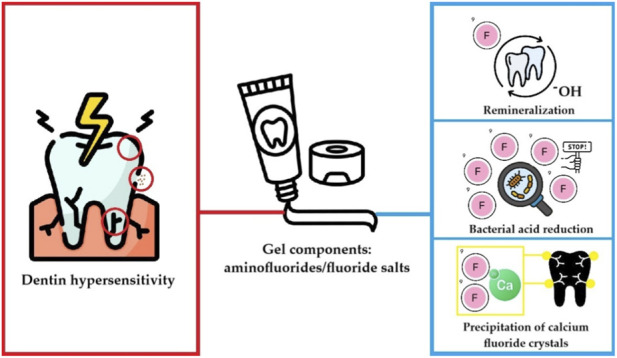
Mechanisms of fluoride gel action in reducing dentine hypersensitivity (created with Freepik.com).

In the domain of medicinal treatments for hypersensitivity, gels containing fluoride are held in high esteem. This phenomenon is primarily attributable to their dual action, which involves the formation of calcium fluoride-like deposits, leading to tubular occlusion. Additionally, these deposits serve to enhance the resistance of enamel and dentin to acid dissolution (Cate, 1999; Dessai et al., 2020). An optimal desensitizing formulation should achieve an initial burst release to provide rapid pain relief, but the key-factor of their long-term effectiveness is the controlled and sustained release of fluoride, which promotes the process of remineralization and the introduction of a layer protecting against acid attack (Cury and Tenuta, 2008; Omidi et al., 2020). However, the fluoride release profile of desensitizing gels can vary significantly depending on their chemical composition, the presence of additional ions, and the physicochemical environment to which the material is exposed (Hicks et al., 2002; Nicholson, 2025). The mode of operation of dental desensitizing gels is presented on Figure 1.

One key environmental factor is the pH of the environment. Low pH promotes the dissolution of fluoride-containing compounds, increasing short-term release but potentially compromising the long-term effectiveness of the material (Arends and Schüthof, 1975; Ten Cate et al., 2006; Morawska-Wilk et al., 2025). By way of contrast, environments that are neutral or slightly alkaline have been found to favor the formation of more stable fluoride-calcium complexes. This, in turn, results in a more controlled release of ions (Malinowski et al., 2012). Recent studies have shown that even small pH fluctuations, due to dietary or bacterial acid effects, can significantly alter fluoride kinetics and tubular occlusion integrity with desensitizing agents (Zhao et al., 2021; Vashisth et al., 2024).

The presence of calcium ions (Ca^2+^) also plays a key role in fluoride dynamics, as calcium can chemically interact with fluoride to form calcium fluoride salt (CaF_2_) or fluorapatite deposits, which contribute to the occlusion of dentinal tubules and the strengthening of mineralized tissue (Larsen and Pearce, 2003; Yang et al., 2017). Moreover, recent *in vitro* findings indicate that calcium-rich environments may also reduce the availability of free fluoride ions in the solution phase, altering diffusion patterns and release kinetics (Di Lauro et al., 2023; Islam et al., 2024). Understanding the balance between precipitation and ion mobility is crucial for optimizing the performance of desensitizing gels, especially in the dynamic conditions of the oral cavity. Constant fluctuations in the pH and ionic composition of saliva affect the bioavailability of desensitizing gel ingredients. Lower pH environments result in more intense ion release, promoting faster release of active compounds. Higher pH and increased salivary flow slow the diffusion process, stabilizing the gel and leading to a more gradual therapeutic effect.

Despite the clinical importance of these interactions, limited data exist on the effect of changes in pH and calcium ion concentration on the free fluoride readings under static *in vitro* conditions of commercial desensitization gels. Previous studies have mostly focused on fluoride release from varnishes or restorative materials, or examined desensitization gels under standardized conditions without simulating changes in the oral environment (Amaechi et al., 1999; Arrais et al., 2004; Aliberti et al., 2025b). To date, only a few recent studies have examined the combined impact of pH and ion concentration on fluoride diffusion and retention. Interestingly, none of these studies focused strictly on desensitizing gels, but mostly on conventional fluoride gels for caries prevention. The formulations of such gels differ from the gels for treatment of hypersensitivity. Therefore, the fluoride release could be governed differently. The aforementioned studies have revealed significant differences that depend on the formulation (Piszko et al., 2025a). These findings highlight the necessity for systematic comparative assessments of commercial formulations under controlled but physiologically relevant conditions. The amount of measurable free fluoride and influence of calcium in incubation medium is reported in literature on various dental biomaterials. Nevertheless, the available data concerning dental desensitizing gels remains restricted. The present study aims to address this paucity of information.

Hence, we hypothesize that measurable fluoride from desensitizing gels is lower in an environment saturated with calcium ions and increases with lowering pH. By simulating environmental conditions representative of fluctuating oral pH and salivary calcium concentrations, this study aims to elucidate how these parameters influence levels of measurable free fluoride which may be potentially translated into remineralization capacity. The results are expected to provide valuable insights into the measurable solution-phase fluoride levels in desensitization gels matrix with potential to enhance their efficacy. Nevertheless, fluoride intake should be carefully monitored, as high concentrations can directly affect enzymatic activity in organisms or induce nephrotoxic effect (Manjunathappa et al., 2023; Małyszek et al., 2025).

## 2 Materials and methods

### 2.1 Incubation solutions

The *in vitro* assays were conducted in twelve distinct liquids. The following substances were utilized in the experiment: tap water, distilled water, demineralized water, 0.9% NaCl solution and artificial saliva (pH = 4.5; 6.0; 7.0; 7.5) with and without the presence of calcium ions. The artificial saliva and sodium chloride solutions were derived from demineralized water. The study utilized two distinct types of artificial saliva: one containing calcium ions and one without. The two formulations were both derived from well-established protocols that are frequently used during *in vitro* studies regarding dental materials (AlMashhadani, 2018; Dobrzyński et al., 2023). The artificial saliva was prepared on the basis of demineralized water by dissolving the mixture of electrolytes and organic components. The following data is presented in Table 1: the source, composition and content of calcium of the utilized incubation solutions. Compositional data is based on the study on fluoride gels reported previously (Piszko et al., 2025a).

**TABLE 1:** Solutions used during experiments: pH, calcium content and composition/origin.

No.	Incubation liquid	pH [a.u.]	Concentration of Ca^2+^ [mg/mL]	Source/Chemical composition
1	Tap water	7.36	0.0730*	Tap water collected at ul. Chałubińskiego 3, wrocław, Poland
2	Distilled water	6.79	0.0	Sourced from laboratory water distiller
3	Demineralized water	6.81	0.0	Stapar (żnin, Poland)
4	NaCl*	7.13	0.0	0.9% NaCl solution based on demineralized water
5	Artificial saliva with Ca^2+^ **	4,5; 6.0; 7.0; 7.5	0.2476	Solution based on demineralized water with addition of urea (1 g/L), NaCl (0.4 g/L), KCl (0.4 g/L), CaCl_2_·2H_2_O (0.908 g/L), NaH_2_PO_4_·2H_2_O (0.78 g/L), and Na_2_S·9H_2_O (0.005 g/L)
6	Artificial saliva without Ca^2+^ **	4,5; 6.0; 7.0; 7.5	0.0	Solution based on demineralized water with addition of urea (1 g/L), NaCl (0.4 g/L), KCl (0.4 g/L), NaH_2_PO_4_·2H_2_O (0.78 g/L), and Na_2_S·9H_2_O (0.005 g/L)

* The calcium ions concentration in tap water was established using Wrocław Municipality Waterworks data (https://www.mpwik.wroc.pl/strefa-klienta/uslugi/uslugi-laboratoryjne/parametry-wody/accessed on 27 October 2025).

** Substances supplied by Chempur (Piekary Śląskie, Poland): sodium chloride (NaCl), potassium chloride (KCl), sodium bisulfate (Na_2_S·9H_2_O), sodium hydrogen phosphate (NaH_2_PO_4_·2H_2_O), and urea. Calcium chloride dihydrate (CaCl_2_·2H_2_O) was manufactured by Sigma Aldrich (St. louis, MO, United States of America).

### 2.2 Investigated desensitizing gels

A study was conducted on two dental desensitizing gels: Teeth Desensitiser TD (LDK GmbH, Frankfurt Oder, Germany) and SensiKIN (Laboratorios KIN, Barcelona, Spain). The evaluated desensitizing dental gels are presented in Supplementary Figure S1. Furthermore, the compositional information is presented in Table 2.

**TABLE 2:** Manufacturers, lot/batch numbers, compositions, and fluoride concentrations of evaluated desensitizing gels.

Gel	Supplier	Lot/Batch number	Composition disclosed by manufacturer	Fluoride source and content
Teeth desensitiser TD	LDK GmbH, frankfurt oder, Germany	TDI10250310	Aqua, glycerin, urea, cellulose gum, potassium nitrate, sodium fluoride, silica, menthol, disodium EDTA	NaF Not disclosed by manufacturer
SensiKIN	Laboratorios KIN, barcelona, Spain	25C01	Sodium fluoride, potassium nitrate	NaF 1,000 ppm

### 2.3 Measurement of pH and free, measurable fluoride

Single time-point fluoride measurement methodology was applied in accordance with the previously published research (Piszko et al., 2025a). Briefly, separate polypropylene tubes were utilized for each gel. In each tube, 0.055 g of desensitizing gel was deposited. Subsequently, 5.5 mL of incubation solution was introduced to the tubes and homogenized using a Vortex V1 (Supplementary Figure S2A, Biosan, Riga, Latvia), and magnetically stirred. Gel-to-solution ratio was based on the previously reported studies on static incubation of dental biomaterials (Garcez et al., 2007; Kosior et al., 2022). The 9,609 Orion electrode (Thermo Scientific, Waltham, MA, United States) wired to CPI-551 processing unit (Supplementary Figure S2B, Elmetron, Zabrze, Poland) was utilized to determine concentration of F^−^ ions. Measurement was recorded immediately following the homogenization process in 5 repetitions (n = 5) and in ambient temperature (24 °C). The methodology applied for measurement of fluoride results in the values of measurable free fluoride in solution.

Before incubation and after the fluoride concentration measurement, the pH values of the incubation solutions were determined using an ESAgP-303W electrode (Eurosensor, Gliwice, Poland) connected to a CPI-505 pH meter (Supplementary Figure S2C, Elmetron). This evaluation was performed in five replicates for each solution (n = 5). In accordance with the recommendation ion selective electrode manufacturer, the calibration was performed prior to the measurement using 2 fluoride standards (1.9 and 19 ppm). The acceptable slope range was 54–60 mV/decade. The electrode slope for calibration curve without TISAB was 59 mV and 58 mV with addition of TISAB.

It should be noted that presented methodology does not concern portions of fluorine retained in the matrix or bound as insoluble salts. Therefore, the aforementioned measurements were repeated with the addition of TISAB II containing CDTA (Thermo Scientific, Waltham, MA, United States) which acts as a ionic strength adjuster and complexing agents liberating bound fluorine. Specifically, 5.5 mL of TISAB II (equivalent to the volume of the incubation solution) was added to each sample prior to homogenization. Fluoride concentration and pH were measured in triplicate (n = 3).

### 2.4 FTIR spectra acquisition

Desensitizing gels were subjected to Attenuated Total Reflectance FTIR analysis (ATR-FTIR). The spectra of SensiKIN and Teeth Desensitiser TD were obtained on FTIR Nicolet 6700 spectrometer (Thermo Scientific, Waltham, MA, United States), equipped with an ATR accessory (Pike Technologies, Fitchburg, MA, United States). The spectra we acquired in 128 scans, resolution of 4 cm^−1^ within the range of 4,000–400 cm^-1^ at 23 °C. The obtained spectra were post-processed and analyzed OriginPro 2025b software (Origin Lab Corporation, Northampton, MA, United States). Processing included baseline subtraction and Savitzky–Golay filter smoothening (polynomial order: 2; points of window: 15).

### 2.5 Statistical methods

The statistical methods used in the presented study were primarily based on an ANOVA test. Multifactorial ANOVA with a Sidak *post hoc* test was used to evaluate the effects of distinct factors and their possible interactions on measurable F- and changes in pH, which are presented as the final pH minus the initial pH. The factors used in the analysis were the manufacturer (type of gel), the presence or absence of Ca^2+^, and the initial pH. In the addition to the statistical significance of the results (p-value), eta squared (ƞ^2^) value was also calculated. This coefficient indicates the strength of the association between the factor and the observed outcome.

The comparison of water and saliva was based on a univariate ANOVA. Water could not be adjusted for fixed Ca^2+^ and pH levels. Therefore, a multifactorial ANOVA could not be performed. Due to the pH of the water samples, the averaged results for artificial saliva with a pH of 7.0 and 7.5 were used to minimize the possible influence of pH on the final result. To ensure the robustness of the multifactorial ANOVA test, the number of measurements in each group being compared (or their combinations) was equal. Q-Q plots were used to confirm the normality of the variables (Supplementary Figure S3). The analysis was performed using R packages: stats (4.1.2), effectsize (1.0.1), emmeans (1.10.5), multcomp (1.4–26) which work within the R statistical environment (R Foundation for Statistical Computing, 2024). Confidence threshold was set to α = 0.001.

## 3 Results

### 3.1 Measurable free fluoride and changes in pH in artificial saliva solutions

While all the tested variables influence F concentration to some extent, initial pH value influences it the most (ANOVA, η^2^ = 0.452; p < 0.001, Table 3). In the vast majority of saliva pairs with the same pH but different concentrations of Ca^2+^, the presence of these ions decreases free measurable F^−^ by 50–140 ppm. Although less pronounced, the effect of other variables is still significant. These variables include the gel manufacturer (η^2^ = 0.187; p < 0.001) and Ca^2+^ presence (η^2^ = 0.092; p < 0.001).

**TABLE 3:** The results of multifactorial ANOVA analysis for data obtained without addition of TISAB. Table summarizes variables, sum of squares, degrees of freedom (df), mean squares, F-statistic (F), p-value (p) and effect size (η^2^). The results with the highest η^2^ are in bold.

Free measurable F^−^ [ppm]						
Variable	Sum of squares	Df	Mean square	F	p	η^2^
Ca^2+^	138,611	1	138,611	30.79	<0.001	0.091
Initial pH	681,458	3	227,153	50.45	<0.001	0.452
Manufacturer	282,031	1	282,031	62.64	<0.001	0.187
Ca^2+^ × initial pH	24,096	3	8,032	1.78	0.159	0.016
Ca^2+^ × manufacturer	1,620	1	1,620	0.36	0.551	0.001
Initial pH × manufacturer	78,531	3	26,177	5.81	0.001	0.052
Ca^2+^ × initial pH × manufacturer	14,568	3	4,856	1.08	0.365	0.010
Residuals	288,140	64	4,502	-	-	-

pH change [a.u.]						
Variable	Sum of squares	Df	Mean square	F	p	η^2^
Ca^2+^	0.03	1	0.03	69.72	<0.001	0.010
Initial pH	3.41	3	1.14	2,272.51	<0.001	0.933
Manufacturer	0.04	1	0.04	89.30	<0.001	0.012
Ca^2+^ × initial pH	0.04	3	0.01	25.34	<0.001	0.010
Ca^2+^ × manufacturer	0.02	1	0.02	42.90	<0.001	0.006
Initial pH × manufacturer	0.06	3	0.02	38.23	<0.001	0.016
Ca^2+^ × initial pH × manufacturer	0.01	3	0.00	9.91	<0.001	0.004
Residuals	0.03	64	0.00	-	-	-

**FIGURE 2:**
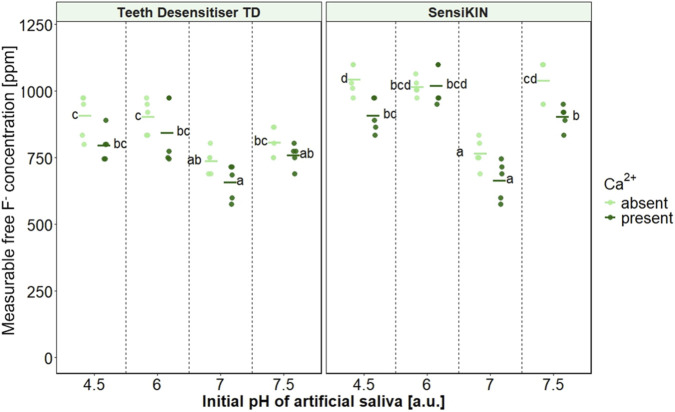
The free measurable fluoride concentration with respect to the composition and pH of the artificial saliva and the type of analyzed gel without addition of TISAB. Mean average is depicted as a horizontal dash. Difference in statistical significance is depicted with different letters (p < 0.001).

Teeth Desensitiser TD gel samples analysis revealed that the presence of Ca^2+^ led to a reduction in free measurable F^−^ across the entire pH range of the artificial saliva solutions: at pH 4.5 from 907 to 796, at pH 6.0 from 903 to 844 ppm, at pH 7.0 from 737 to 658 and at pH 7.5 from 807 to 759 ppm. A similar trend was observed for SensiKIN samples (except pH = 6.0), where measurable free fluoride level decreased at pH 4.5 from 1,043 to 908 ppm, at pH 7.0 from 766 to 665 ppm and at pH 7.5 from 1,040 to 903 ppm. Therefore, highest value of measurable fluoride obtained in artificial saliva solutions was 1,043 ppm for SensiKIN at pH = 4.5 in solution without addition of calcium ions, and lowest value (658 ppm) was observed for Teeth Desensitiser TD gel at pH = 7.0 with addition of Ca^2+^. Figure 2 shows plot depicting the concentration of fluoride ions in artificial saliva at different pH levels and with and without presence of Ca^2+^.

**FIGURE 3:**
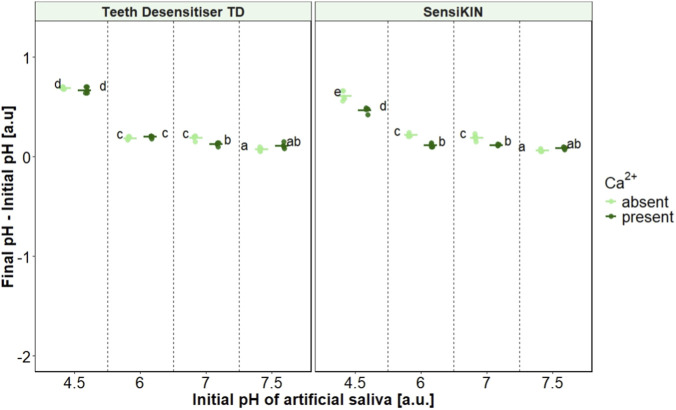
Changes in pH, described as the difference between the initial and final pH, after the administration of the evaluated gels without addition of TISAB. Mean average is depicted as a horizontal dash. Difference in statistical significance is depicted with different letters (p < 0.001).

The final pH value is affected the most by initial pH value (η^2^ = 0.933; p < 0.001) (Table 3). For Teeth Desensitiser TD the only statistically significant change occurred at pH = 7.0. Nevertheless, the difference was 0.064 a.u. (Figure 3). For SensiKIN statistically significant changes occurred at pH = 4.5; 6.0 and 7.0 with the difference 0.144; 0.104 and 0.074 respectively.

**FIGURE 4:**
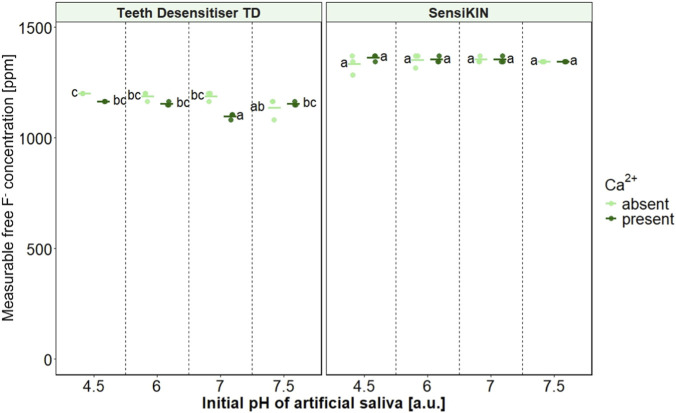
The free measurable fluoride concentration with respect to the composition and pH of the artificial saliva and the type of analyzed gel with addition of TISAB. Mean average is depicted as a horizontal dash. Difference in statistical significance is depicted with different letters (p < 0.001).

As per measured free fluoride in the samples with addition of TISAB (Figure 4), they were higher in comparison to measurement without addition of ionic strength adjuster and chelating agent by approximately 359 ppm on average for Teeth Desensitiser TD and 429 ppm on average for SensiKIN. For Teeth Desensitiser TD, the reported measurable free fluoride were statistically insignificant, except for artificial saliva with pH = 6.0, where the value was 91.7 ppm higher for solution without calcium. The values obtained for SensiKIN were all statistically significant between each other depicting almost no variability. According to multifactorial ANOVA (Table 4), measurable fluoride concentration mostly affected by gel composition (η^2^ = 0.933; p < 0.001).

**TABLE 4:** The results of multifactorial ANOVA analysis for data obtained with addition of TISAB. Table summarizes variables, sum of squares, degrees of freedom (df), mean squares, F-statistic (F), p-value (p) and effect size (η^2^). The results with the highest η^2^ are in bold.

Free measurable F^−^ [ppm]						
Variable	Sum of squares	Df	Mean square	F	p	η^2^
Ca^2+^	2,338	1	2,338	5.10	0.031	0.005
Initial pH	3,506	3	1,169	2.55	0.073	0.007
Manufacturer	428,463	1	428,463	934.83	<0.001	0.915
Ca^2+^ × initial pH	5,002	3	1,667	3.64	0.023	0.011
Ca^2+^ × manufacturer	5,526	1	5,526	12.06	0.002	0.012
Initial pH × manufacturer	3,668	3	1,223	2.67	0.064	0.008
Ca^2+^ × initial pH × manufacturer	4,956	3	1,652	3.60	0.024	0.011
Residuals	14,667	32	458	-	-	-

pH change [a.u.]						
Variable	Sum of squares	Df	Mean square	F	p	η^2^
Ca^2+^	0.0008	1	0.0008	0.81	0.374	0.000
Initial pH	62.3299	3	20.7766	22,461.22	<0.001	0.999
Manufacturer	0.0000	1	0.0000	0.02	0.888	0.000
Ca^2+^ × initial pH	0.0008	3	0.0003	0.28	0.840	0.000
Ca^2+^ × manufacturer	0.0004	1	0.0004	0.38	0.542	0.000
Initial pH × manufacturer	0.0001	3	0.0001	0.05	0.985	0.000
Ca^2+^ × initial pH × manufacturer	0.0027	3	0.0009	0.96	0.422	0.000
Residuals	0.0296	32	0.0009	-	-	-

**FIGURE 5:**
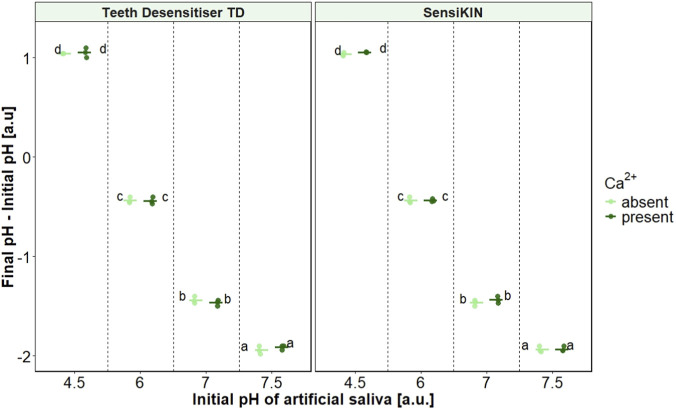
Changes in pH, described as the difference between the initial and final pH, after the administration of the evaluated gels with addition of TISAB. Mean average is depicted as a horizontal dash. Difference in statistical significance is depicted with different letters (p < 0.001).

As presented in Table 4, the final pH is affected only and most prominently by initial pH (η^2^ = 0.999; p < 0.001). There are no statistical significances between solutions with the same pH. Final measured pH value of all solutions is approximately constant and equal to 5.55 regardless of initial pH (Figure 5).

### 3.2 Comparison between artificial saliva and reference aqueous incubation media

**FIGURE 6:**
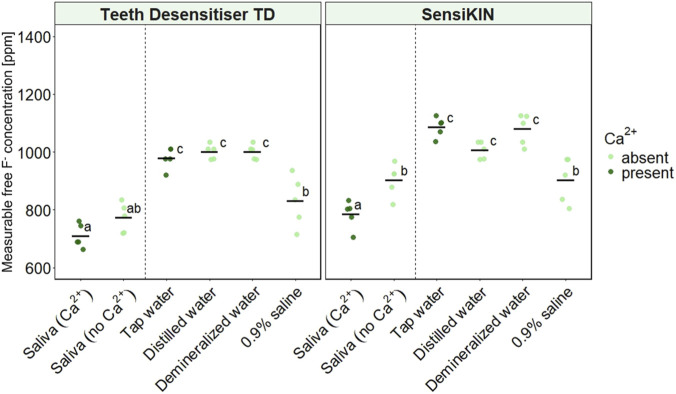
A comparison of free, measurable F in artificial saliva with a pH of 7.0–7.5 and in different types of water was conducted for samples without addition of TISAB. Tap water was marked as containing Ca^2+^ ions, while the others were marked as devoid of these ions. Mean average is depicted as a horizontal dash. Difference in statistical significance is depicted with different letters (p < 0.001).

Since the water samples used in the current study could not be adjusted for pH and calcium content, a comparison of water and artificial saliva samples was made only for fluoride levels. The general observation is that tap, distilled and demineralized water samples tend to exhibit greater and statistically significant measurable free fluoride in comparison with artificial saliva specimens (Figure 6). For the 0.9% saline solution, the concentration was comparable to saliva without Ca^2+^.

**FIGURE 7:**
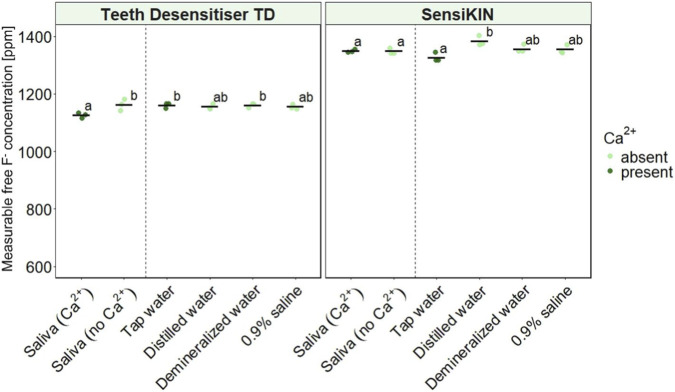
A comparison of free, measurable F in artificial saliva with a pH of 7.0–7.5 and in different types of water was conducted for samples with addition of TISAB. Tap water was marked as containing Ca^2+^ ions, while the others were marked as devoid of these ions. Mean average is depicted as a horizontal dash. Difference in statistical significance is depicted with different letters (p < 0.001).

Figure 7 compares combined saliva samples with water-based samples with addition of TISAB. The level of variability is less pronounced as compared with pathway omitting addition of TISAB. Levels of measurable free fluoride are comparable for all presented solutions within the same gel sample. Nevertheless, SensiKIN resulted in greater values following the trend presented on Figure 4.

### 3.3 FTIR spectra of desensitizing gels

**FIGURE 8:**
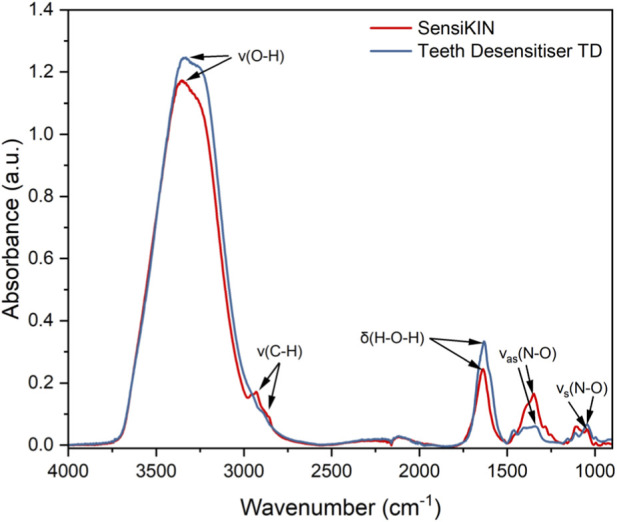
FTIR spectra of SensiKIN and Teeth Desentisiser TD gels with identified bands assigned on the plot.

For additional structural information, FTIR spectra of SensiKIN and Teeth Desentisiser TD desensitizing gels were acquired and presented on Figure 8. Presented results support structural confirmation of the gels substantiating their reliability. The spectra exhibited similar patterns and allowed for assignment of following characteristic absorption bands indicative of its functional groups and molecular structure. A broad peak at 3,344 cm^-1^ corresponded to the O-H stretching vibration (ν(O−H)), suggesting the presence of hydroxyl groups or water in hydrogel matrix (Dashnau et al., 2006). Aliphatic C-H stretching vibrations (ν(C−H)) appeared at 2,924 cm^-1^ and 2,859 cm^-1^, which could be attributable to methylene groups in glycerol or other polymer-based additives (Danish et al., 2017). The bending mode of water molecules, δ(H-O-H), was observed at 1,635 cm^-1^ (Dashnau et al., 2006), while the asymmetric stretching (ν_as_ (N−O)) and symmetric stretching (ν_s_ (N−O) of the nitrate group were identified at 1,351 cm^-1^ and 1,043 cm^-1^, respectively, confirming nitrate incorporation (Kumar et al., 2019). Presence of nitrate is in line with the composition disclosed by manufacturers Table 2, where potassium nitrate is disclosed. NaF is an ionic salt that lacks covalent bonds capable of producing infrared-active vibrations, and thus it does not generate a characteristic FTIR spectrum. Consequently, presence of fluoride containing ingredient was confirmed through a fluoride concentration assay using ion-selective electrodes in the presented *in vitro* study.

## 4 Discussion

The obtained results demonstrate that measurable free fluoride levels in solution are influenced by the pH of the environment, as also concluded in the previously reported studies conducted on restorative dental materials (Aliberti et al., 2025c; 2025a). It was established that significantly greater amounts of fluoride were observed under acidic conditions for both Teeth Desensitiser TD and SensiKIN desensitizing gels. The maximum concentration for both materials was observed at a pH of 4.5, which resembles the environment commonly encountered in the oral cavity following acidogenic challenges, such as after food intake or early enamel demineralization (Atkinson et al., 2021). As indicated by the findings of earlier studies on fluoride gels and nanohydroxyapatite hydrogels, this phenomenon can be attributed to the heightened solubility and permeability of gel matrices in acidic conditions. Protonation weakens intermolecular hydrogen bonding and ionic interactions within the polymer network, causing structural loosening, increased swelling, and higher matrix permeability. This, in turn, promotes accelerated ion diffusion and exchange with the surrounding medium (Wiglusz et al., 2023; Piszko et al., 2025a). In such cases, the presence of hydrogen ions therefore acts as a trigger that disrupts or softens the gel matrix, enhancing fluoride mobility and enabling more efficient transport into saliva or onto enamel surfaces. Furthermore, this mechanism is indicative of a protective adaptation, analogous to the natural defensive response of saliva. In this response, periods of lower pH trigger processes that are aimed at restoring mineral balance (Turska-Szybka et al., 2024).

The pH-dependent behavior aligns measurable fluoride concentration with the conditions under which teeth are most susceptible to demineralization. It is hypothesized that enhanced fluoride burst in a slightly acidic environment may play a significant role in promoting remineralization and preventing mineral loss in the early stages of caries, when superficial enamel is dissolved (Piszko et al., 2025b). It is especially relevant after the intake of fermentable carbohydrates, when plaque pH falls rapidly and salivary buffering may be insufficient to immediately counter the acid challenge. The resulting accelerated fluoride burst promotes fluorapatite formation, which in turn reinforces enamel resistance and contributes to the early arrest of demineralization (Anu et al., 2019; Polyakova et al., 2024). Such pH responsive fluoride concentrations mirrors the cariostatic behavior observed in many therapeutic materials.

Compared with the prophylaxis gels examined in our previous study, the evaluated NaF-based desensitizing gels differ substantially in their free fluoride concentrations and environmental responsiveness. Prophylaxis gels, which contained higher fluoride concentrations and structurally denser matrices, containing substances such as xanthan gum or magnesium aluminum silicate, showed maximal fluoride concentration under neutral to slightly alkaline conditions and were less responsive to mild acidification (Piszko et al., 2025a). In contrast, the desensitizing gels in the present study demonstrate increased measurable solution-phase fluoride concentrations in acidic pH. Although, over neutral pH, at base levels the concentrations were also elevated. This behavior likely reflects the influence of formulation excipients specific to desensitizing products, such as potassium nitrate, urea, and cellulose gum, which reduce cross-link density, enhance gel hydration, and promote proton-mediated matrix softening. Collectively, these findings highlight that desensitizing gels constitute a mechanistically distinct category of fluoride-delivering materials, optimized for rapid ion burst under cariogenic pH fluctuations, thereby justifying their evaluation in a dedicated study. At pH values that are neutral or close to neutral, which are characteristic of a healthy oral environment, free fluoride readings under static *in vitro* conditions becomes more gradual. This allows for the maintenance of a fluoride concentration that supports continuous tissue remineralization and also promotes the reduction of hypersensitivity (Lynch et al., 2004; Markowitz, 2013). The observed measurable solution-phase free fluoride under standardized *in vitro* condition can be perceived as favorable, as they provide a free fluoride delivery system that responds to fluctuations in oral pH, offering intensified concentration during acid attacks and equalized single time-point solution-phase free fluoride levels in physiological conditions. The observed effects, might have potential impact in enhancing the efficacy of these gels in caries prevention and management of dentin hypersensitivity (Koehler et al., 2024). Nevertheless, the presented measurable free fluoride data were registered as a static, single time-point study and prolonged data shall confirm the plausible clinical significance.

Literature reports multiple studies on measurable free fluoride concentration from dental preparations, including gels and varnishes. These studies have identified several factors influencing this process. For example, the variety of matrix in which fluoride is present. Preparations based on amine fluorides do not interact with other ions as rapidly as fluoride ions available from formulations containing NaF or KF. This is due to the fact that the fluoride ion in amine fluorides is covalently bound, which influences the dissociation mechanism. Free fluoride combines more readily with cations such as calcium, causing calcium fluoride precipitation. This clearly has a reported significant impact on the final free fluoride concentration in the solution (Kosior et al., 2017; Morawska-Wilk et al., 2025). However, it is worth noting and considering that in conditions where the medium does not contain calcium, the presence of other ions, such as chloride and acetate, might facilitate the availability of fluoride ions from NaF or KF-based preparations. This phenomenon results from the interaction of sodium cations, for example, with Cl anions, which leads to increased levels of fluoride concentration (Ralph Rawls, 1986). Another important factor is the composition of the gel matrix. The tested SensiKIN and Teeth Desensitiser TD gels contain fluoride in the form of NaF. Despite the fact that the gels were subjected to identical incubation conditions, with the same fluids and parameters, the quantity of free, measurable fluoride ions concentration differed. This finding serves to contribute to the hypothesis that the initial concentration and supplementary excipients contained within the gels are capable of exerting a modulatory effect on the measurable free fluoride in solution (Turkalj et al., 2020; Turska-Szybka et al., 2024).

One of the principal objectives of the present study was to evaluate the impact of calcium on the measurable concentration of fluoride in the tested NaF-based desensitizing gels. It has been demonstrated by preceding studies that elevated fluoride concentrations are discernible in deionized water solutions in comparison to artificial saliva. Evidently, the presence of cations and anions in saliva exerts a substantial influence on the concentration of free fluoride (Levallois et al., 1998; Kosior et al., 2022). Presented results demonstrate that calcium significantly reduces content of measurable free fluoride. Regardless of saliva pH, the addition of calcium resulted in the reduced concentration of fluoride than in saliva without calcium. One possible explanation for the decrease in free fluoride readings from the applied gel in saliva with higher calcium content is the formation of calcium fluoride (CaF_2_). This phenomenon, reported in literature, affects the production of substances in the dentinal tubules, such as calcium fluoride or fluorapatite, which are intended to reduce hypersensitivity (Ramli et al., 2022). The observed increase in measurable free fluoride after addition of TISAB/CDTA may be consistent with the presence of complexed or otherwise non-free fluoride species. However, the present study did not directly identify the chemical form of bound fluoride or confirm CaF_2_ precipitation. Finally, the level of measurable free fluoride in the evaluated gels after homogenization are within the range of cariostatic and remineralization threshold reported in literature, which are in the range of 250–1,500 ppm on average (Damato et al., 1990; Yeh et al., 2025).

The fluoride concentration in the 0.9% NaCl solution was comparable to calcium-free saliva. In this case, the ionic strength of the solution may be the decisive factor. Both Ca^2+^ ions and saline ions cause higher osmolality, which might influence the flow and mobility of fluoride ions into the medium. A similar relationship was observed in previously reported study (Piszko et al., 2025a). Of three gels investigated in the referenced study with identical fluoride content, lower measurable fluoride levels were observed in calcium-free saliva than in deionized water. This could reflect that calcium presence might limit the measurable free fluoride concentration by not only leading to CaF_2_ precipitation, but also by inhibiting its measurable concentration possibly from the gel into saliva through the increase in ionic strength by other ions. It is hence hypothesized that a hypotonic environment might favor increased concentrations of measurable solution-phase fluoride. This statement is further supported by increased and comparable concentrations after treatment with ionic strength adjuster such as TISAB.

## 5 Conclusion

This preliminary *in vitro* study showed that pH, calcium-containing medium, gel composition, and TISAB/CDTA addition influenced measurable free fluoride detected in solution immediately after homogenization under static single time-point conditions. The highest concentration of measurable free fluoride in solution was observed at slightly acidic pH. Conversely, decreased levels of measurable solution-phase free fluoride were denoted at the neutral pH (prevalent in the oral cavity). These findings may inform future studies evaluating whether measured solution-phase fluoride levels translate into remineralization or desensitizing outcomes. The presence of calcium in saliva has been shown to reduce the concentration of solution-phase measurable free fluoride. This outcome might be investigated in the future works to explore curtailing the cariostatic potential of the formulation. Furthermore, calcium is not the only factor influencing measurable fluoride levels from the two gels tested. The investigation revealed that the 0.9% NaCl solution had a significant effect on level of measurable solution-phase fluoride. This suggests that ionic strength may influence the fluoride mobility. Given above, research regarding future desensitizing gels formulations might take into consideration whether addition of complexing or buffering agents influence measurable solution-phase free fluoride under standardized *in vitro* conditions. FTIR allowed for insight into the structure of gels. These findings should be interpreted as solution-phase free fluoride measurements and do not establish fluoride release kinetics, sustained release behavior, biological or clinical bioavailability. The limitation of the presented study includes evaluation of just two commercial formulations, static incubation and single time-point measurement of free measurable fluoride. Future scope might include dynamic salivary stimulation and cyclic alterations of pH.

## Data Availability

The raw data supporting the conclusions of this article will be made available by the authors, without undue reservation.

## References

[B1] Aliberti A. Di Duca F. Triassi M. Montuori P. Scippa S. Piscopo M. (2025a). The effect of different pH and temperature values on Ca2+, F−, PO43−, OH−, Si, and Sr2+ release from different bioactive restorative dental materials: an *in vitro* study. Polym. (Basel) 17, 640. 10.3390/polym17050640 40076132 PMC11902544

[B2] Aliberti A. Garcia-Godoy F. Borges A. L. S. Tribst J. P. M. Gasparro R. Mariniello M. (2025b). Calcium, phosphate and fluoride ionic release from dental restorative materials for elderly population: an *in vitro* analysis. Front. Oral Health 6, 1609502. 10.3389/froh.2025.1609502 40860792 PMC12375625

[B3] Aliberti A. Gasparro R. Triassi M. Piscopo M. Ausiello P. Tribst J. P. M. (2025c). Fluoride release from pediatric dental restorative materials: a laboratory investigation. Dent. J. (Basel). 13, 224. 10.3390/dj13050224 40422644 PMC12110631

[B4] AlMashhadani H. A. (2018). Study the effect of Punica granatum as oral antifungal on the corrosion inhibition of dental amalgam alloy in saliva. J. Mater. Environ. Sci. 9, 662–671. 10.26872/jmes.2018.9.2.73

[B5] Amaechi B. T. Higham S. M. Edgar W. M. (1999). Factors influencing the development of dental erosion *in vitro:* Enamel type, temperature and exposure time. J. Oral Rehabil. 26, 624–630. 10.1046/j.1365-2842.1999.00433.x 10447814

[B6] Anu V. Madan Kumar P. Shivakumar M. (2019). Salivary flow rate, pH and buffering capacity in patients undergoing fixed orthodontic treatment – a prospective study. Indian J. Dent. Res. 30, 527–530. 10.4103/ijdr.IJDR_74_16 31745047

[B7] Arends J. Schüthof J. (1975). Fluoride content in human enamel after fluoride application and washing – an *in vitro* study. Caries Res. 9, 363–372. 10.1159/000260178 1055639

[B8] Arrais C. A. G. Chan D. C. N. Giannini M. (2004). Effects of desensitizing agents on dentinal tubule occlusion. J. Appl. Oral Sci. 12, 144–148. 10.1590/S1678-77572004000200012 21365138

[B9] Atkinson F. S. Khan J. H. Brand-Miller J. C. Eberhard J. (2021). The impact of carbohydrate quality on dental plaque pH: does the glycemic index of starchy foods matter for dental health? Nutrients 13, 2711. 10.3390/nu13082711 34444871 PMC8401118

[B10] Bartold P. M. (2006). Dentinal hypersensitivity: a review. Aust. Dent. J. 51, 212–218. quiz 276. 17037886

[B11] Brännström M. (1986). The hydrodynamic theory of dentinal pain: sensation in preparations, caries, and the dentinal crack syndrome. J. Endod. 12, 453–457. 10.1016/S0099-2399(86)80198-4 3465849

[B12] Cate J. M. ten (1999). Current concepts on the theories of the mechanism of action of fluoride. Acta Odontol. Scand. 57, 325–329. 10.1080/000163599428562 10777135

[B13] Costa R. S. A. Rios F. S. Moura M. S. Jardim J. J. Maltz M. Haas A. N. (2014). Prevalence and risk indicators of dentin hypersensitivity in adult and elderly populations from Porto alegre, Brazil. J. Periodontol. 85, 1247–1258. 10.1902/jop.2014.130728 24527854

[B14] Cury J. A. Tenuta L. M. A. (2008). How to maintain a cariostatic fluoride concentration in the oral environment. Adv. Dent. Res. 20, 13–16. 10.1177/154407370802000104 18694871

[B15] Damato F. A. Strang R. Stephen K. W. (1990). Effect of fluoride concentration on remineralization of carious enamel an *in vitro* pH-Cycling study. Caries Res. 24, 174–180. 10.1159/000261262 2364402

[B16] Danish M. Mumtaz M. W. Fakhar M. Rashid U. (2017). Response surface methodology based optimized purification of the residual glycerol from biodiesel production process. Chiang Mai J. Sci. 44 (4), 1570–1582.

[B17] Dashnau J. L. Nucci N. V. Sharp K. A. Vanderkooi J. M. (2006). Hydrogen bonding and the cryoprotective properties of glycerol/water mixtures. J. Phys. Chem. B 110, 13670–13677. 10.1021/JP0618680 16821896

[B18] Davari A. Ataei E. Assarzadeh H. (2013). Dentin hypersensitivity: etiology, diagnosis and treatment; a literature review. J. Dent. (Shiraz) 14, 136–145. 24724135 PMC3927677

[B19] Dessai A. Shetty N. Srikant N. (2020). Evaluation of the effectiveness of fluoridated and non-fluoridated desensitizing agents in dentinal tubule occlusion using scanning electron microscopy. An *in-vitro* study. Dent. Res. J. (Isfahan). 17, 193–199. 32774796 PMC7386371

[B20] Di Lauro A. Di Duca F. Montuori P. Dal Piva A. M. de O. Tribst J. P. M. Borges A. L. S. (2023). Fluoride and calcium release from alkasite and glass ionomer restorative dental materials: *in vitro* study. J. Funct. Biomater. 14, 109. 10.3390/jfb14020109 36826908 PMC9967494

[B21] Dionysopoulos D. Gerasimidou O. Beltes C. (2023). Dentin hypersensitivity: etiology, diagnosis and contemporary therapeutic approaches—A review in literature. Appl. Sci. 13, 11632. 10.3390/app132111632

[B22] Dobrzyński W. Nikodem A. Diakowska D. Wiglusz R. J. Watras A. Dobrzyński M. (2023). Comparison of the fluoride ion release from nanofluoroapatite-modified orthodontic cement under different pH conditions - an *in vitro* study. Acta Bioeng. Biomech. 25, 159–176. 10.37190/ABB-02321-2023-02

[B23] Garcez R. M. V. de B. Buzalaf M. A. R. de Araújo P. A. (2007). Fluoride release of six restorative materials in water and pH-cycling solutions. J. Appl. Oral Sci. 15, 406–411. 10.1590/s1678-77572007000500006 19089169 PMC4327260

[B24] Gillam D. G. Seo H. S. Bulman J. S. Newman H. N. (1999). Perceptions of dentine hypersensitivity in a general practice population. J. Oral Rehabil. 26, 710–714. 10.1046/j.1365-2842.1999.00436.x 10520145

[B25] Hicks J. Garcia-Godoy F. Donly K. Flaitz C. (2002). Fluoride-releasing restorative materials and secondary caries. Dent. Clin. North Am. 46, 247–276. 10.1016/S0011-8532(01)00004-0 12014034

[B26] Manjunathappa T. Devegowda D. Mysore N. K. Vishwanath P. (2023). Association between drinking water fluoride and the serum alkaline phosphatase and phosphate levels in pregnant women and newborn infants. Dent. Med. Probl. 60, 569–575. 10.17219/dmp/132692 37555277

[B27] Islam M. S. Padmanabhan V. Abry M. F. Mousa Ahmed K. M. Aryal A C S. Rahman M. M. (2024). The impact of pre-treatment with desensitizing agents on the effectiveness of In-Office bleaching: an *in vitro* study. Materials 17, 6097. 10.3390/ma17246097 39769701 PMC11678487

[B28] Koehler J. Ramakrishnan A. N. Ludtka C. Hey J. Kiesow A. Schwan S. (2024). The influence of oral cavity physiological parameters: temperature, pH, and swelling on the performance of denture adhesives - *in vitro* study. BMC Oral Health 24, 206. 10.1186/s12903-024-03967-7 38336698 PMC10858572

[B29] Kosior P. Dobrzyński M. Korczyński M. Herman K. Czajczyńska-Waszkiewicz A. Kowalczyk-Zając M. (2017). Long-term release of fluoride from fissure sealants—In vitro study. J. Trace Elem. Med. Biol. 41, 107–110. 10.1016/J.JTEMB.2017.02.014 28347456

[B30] Kosior P. Dobrzynski M. Zakrzewska A. Diakowska D. Nienartowicz J. Blicharski T. (2022). Comparison of the fluoride ion release from composite and compomer materials under varying pH conditions—preliminary *in vitro* study. Appl. Sci. 12, 12540. 10.3390/app122412540

[B31] Kumar J. S. John V. S. Rajini P. S. P. (2019). Optical and mechnical studies of potassium nitrate (KNO3) single crystal. JETIR1901468 J. Emerg. Technol. Innovative Res. 6, 549–556.

[B32] Larsen M. J. Pearce E. I. F. (2003). Saturation of human saliva with respect to calcium salts. Arch. Oral Biol. 48, 317–322. 10.1016/S0003-9969(03)00007-4 12663077

[B33] Levallois B. Fovet Y. Lapeyre L. Gal J. Y. (1998). *In vitro* fluoride release from restorative materials in water versus artificial saliva medium (SAGF). Dent. Mater. 14, 441–447. 10.1016/S0300-5712(99)00019-6 10483407

[B34] Lynch R. M. Navada R. Walia R. (2004). Low-levels of fluoride in plaque and saliva and their effects on the demineralisation and remineralisation of enamel; role of fluoride toothpastes. Int. Dent. J. 54, 304–309. 10.1111/j.1875-595X.2004.tb00003.x 15509081

[B35] Malinowski M. Duggal M. S. Strafford S. M. Toumba K. J. (2012). The effect of varying concentrations of fluoridated milk on enamel remineralisation *in vitro* . Caries Res. 46, 555–560. 10.1159/000341220 22922508

[B36] Małyszek A. Zawiślak I. Kulus M. Watras A. Kensy J. Kotela A. (2025). Assessment of fluoride intake risk via infusions of commercial leaf teas available in Poland using the target hazard quotient index approach. Foods 14, 2944. 10.3390/foods14172944 40941060 PMC12428000

[B37] Markowitz K. (2013). A new treatment alternative for sensitive teeth: a desensitizing oral rinse. J. Dent. 41, S1–S11. 10.1016/j.jdent.2012.09.007 23000522

[B38] Morawska-Wilk A. Kensy J. Kiryk S. Kotela A. Kiryk J. Michalak M. (2025). Evaluation of factors influencing fluoride release from dental nanocomposite materials: a systematic review. Nanomaterials 15, 651. 10.3390/nano15090651 40358267 PMC12073368

[B39] Nicholson J. W. (2025). Stannous fluoride in toothpastes: a review of its clinical effects and likely mechanisms of action. J. Funct. Biomater. 16, 73. 10.3390/jfb16030073 40137352 PMC11942899

[B40] Omidi S. Ahadian A. Hadidi G. Mousavi S. J. Forghani M. (2020). Evaluation of dentin adaptability of fluoride varnish as a root canal sealer using scanning electron microscopy. Front. Dent. 16, 335–341. 10.18502/fid.v16i5.2278 32123873 PMC7040556

[B41] Piszko P. J. Kulus M. Piszko A. Kiryk J. Kiryk S. Kensy J. (2025a). The influence of calcium ions and pH on fluoride release from commercial fluoride gels in an *in vitro* study. Gels 11, 486. 10.3390/gels11070486 40710648 PMC12296090

[B42] Piszko P. J. Piszko A. Kiryk S. Kiryk J. Kensy J. Michalak M. (2025b). Fluoride release from two commercially available dental fluoride gels—in vitro study. Gels 11, 135. 10.3390/gels11020135 39996678 PMC11854721

[B43] Polyakova M. Egiazaryan A. Doroshina V. Zaytsev A. Malashin A. Babina K. (2024). The effect of oral care foams and a spray on salivary pH changes after exposure to acidic beverages in young adults. Dent. J. (Basel). 12, 93. 10.3390/dj12040093 38668005 PMC11049306

[B44] Porto I. C. C. M. Andrade A. K. M. Montes M. A. J. R. (2009). Diagnosis and treatment of dentinal hypersensitivity. J. Oral Sci. 51, 323–332. 10.2334/josnusd.51.323 19776498

[B45] R Foundation for Statistical Computing (2024). R: a language and environment for statistical computing. Available online at: https://www.R-project.org/(Accessed October 22, 2025).

[B46] Ralph Rawls H. (1986). Fluoride-releasing acrylics. J. Biomater. Appl. 1, 382–405. 10.1177/088532828600100406 3333407

[B47] Ramli R. Ghani N. Taib H. Mat Baharin N. H. (2022). Successful management of dentin hypersensitivity: a narrative review. Dent. Med. Probl. 59, 451–460. 10.17219/dmp/143354 36206495

[B48] ten Cate J. M. Exterkate R. A. M. Buijs M. J. (2006). The relative efficacy of fluoride toothpastes assessed with pH cycling. Caries Res. 40, 136–141. 10.1159/000091060 16508271

[B49] Turkalj M. Šutej I. Peroš K. (2020). Comparison of fluoride ion release from fluoride gel in various solvents. Acta Stomatol. Croat. 54, 147–154. 10.15644/ASC54/2/4 32801373 PMC7362733

[B50] Turska-Szybka A. Piotrkowicz Z. Prokopczyk M. Olczak-Kowalczyk D. Sierakowski M. Gozdowski D. (2024). Concentration of fluoride in saliva after fluoride gel application: a randomised clinical trial. Int. Dent. J. 74, 794–800. 10.1016/j.identj.2024.01.005 38734515 PMC11287168

[B51] Vashisth P. Naik S. Sharma S. Murry J. N. Singh V. Chandak A. (2024). Comparative evaluation of longevity of fluoride release from three different fluoride varnishes: an observational study. Int. J. Clin. Pediatr. Dent. 17, 341–345. 10.5005/jp-journals-10005-2778 39144504 PMC11320789

[B52] Wiglusz K. Dobrzynski M. Gutbier M. Wiglusz R. J. (2023). Nanofluorapatite hydrogels in the treatment of dentin hypersensitivity: a study of physiochemical properties and fluoride release. Gels 9, 271. 10.3390/gels9040271 37102883 PMC10137577

[B53] Yang J.-C. Hu H.-T. Lee S.-Y. Hsieh S.-C. Huang P.-C. Ma C.-F. (2017). *In vitro* evaluation of dentin tubule occlusion for novel calcium lactate phosphate (CLP) paste. Materials 10, 228. 10.3390/ma10030228 28772594 PMC5503312

[B54] Yeh C.-H. Wang Y.-L. Vo T. T. T. Lee Y.-C. Lee I.-T. (2025). Fluoride in dental caries prevention and treatment: mechanisms, clinical evidence, and public health perspectives. Healthc. (Basel) 13, 2246. 10.3390/healthcare13172246 40941599 PMC12427920

[B55] Zhang M. Ma Y. Ye X. Zhang N. Pan L. Wang B. (2023). TRP (transient receptor potential) ion channel family: structures, biological functions and therapeutic interventions for diseases. Signal Transduct. Target. Ther. 8, 261. 10.1038/s41392-023-01464-x 37402746 PMC10319900

[B56] Zhao X. Wang L. Pan J. Malmstrom H. Ren Y.-F. (2021). Effects of desensitizing dentifrices on dentin tubule occlusion and resistance to erosive challenges. BMC Oral Health 21, 610. 10.1186/s12903-021-01977-3 34847898 PMC8638163

