# Supplementary material for: pH- and calcium-dependent solution-phase free fluoride in dental desensitizing gels: a preliminary in vitro study
Source: Front Bioeng Biotechnol. 2026 Jul 10;14:1788855. doi: 10.3389/fbioe.2026.1788855 (PMC13395941; doi:10.3389/fbioe.2026.1788855)
Supplement: Supplementary file 1 [file Supplementaryfile1.docx]

Supplementary Material

# Supplementary Data


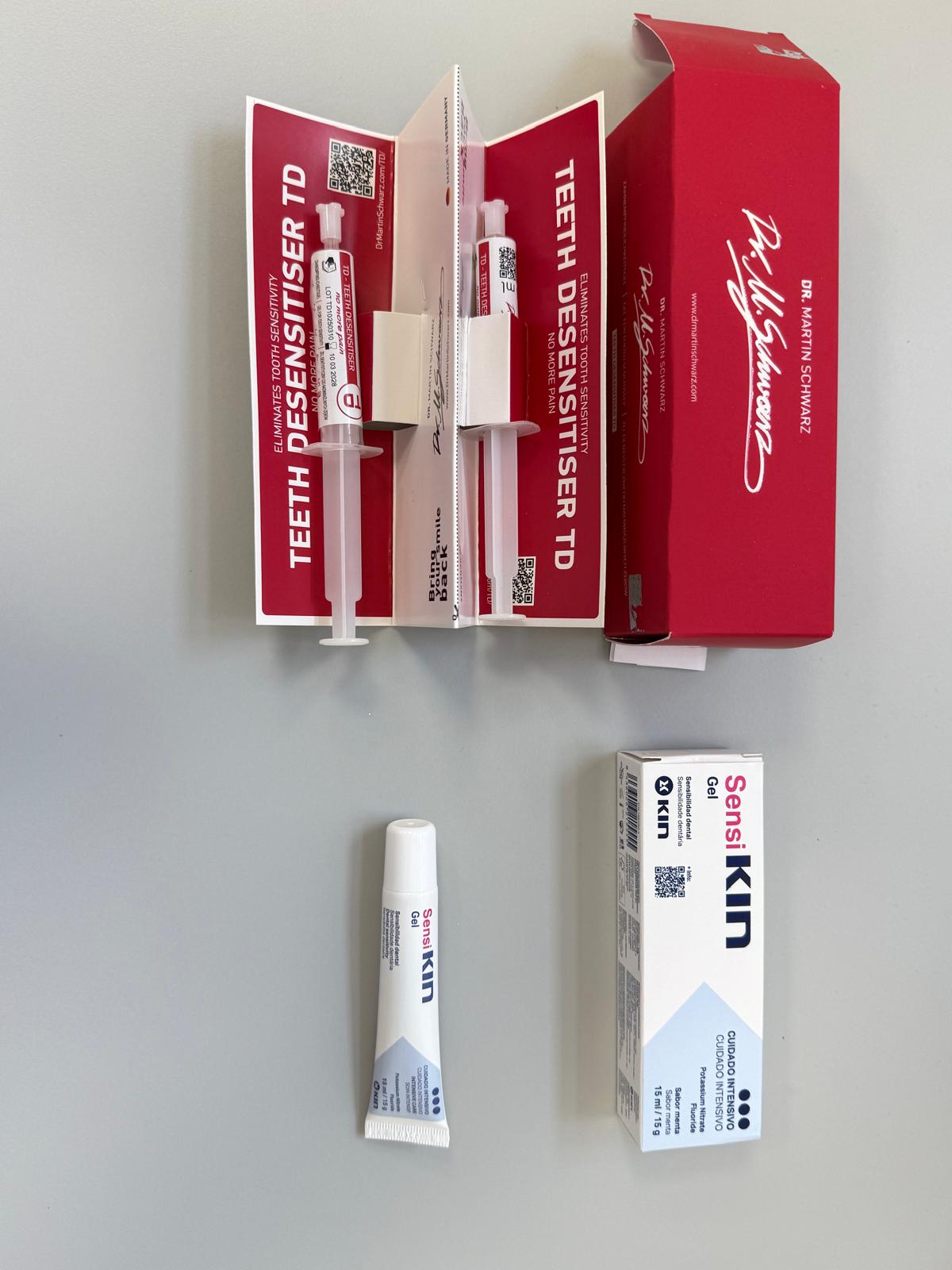


**Supplementary Figure 1.** Teeth Desensitiser TD and SensiKIN - commercial desensitizing gels.


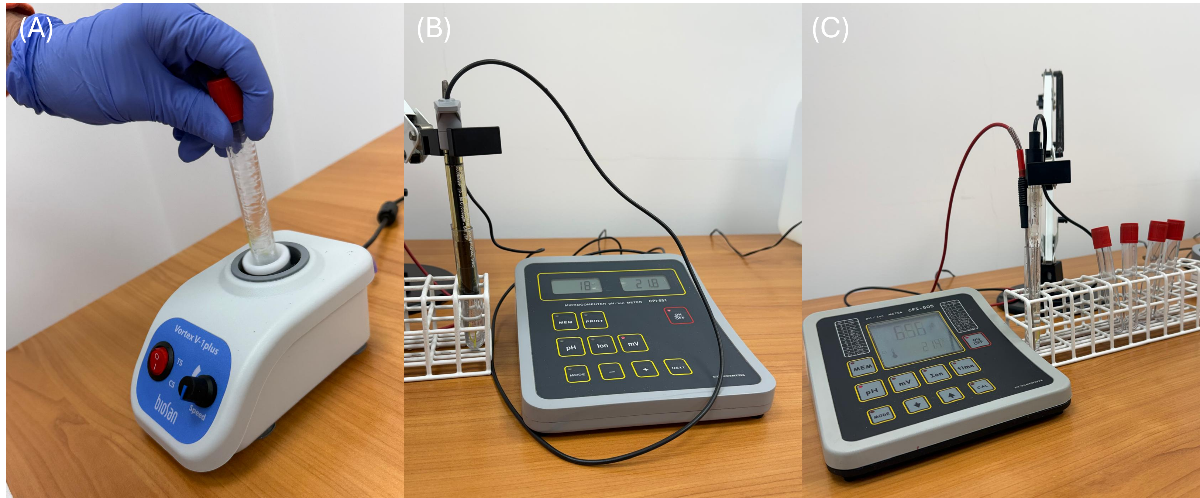


**Supplementary Figure 2.** Homogenization of samples (A), ion-selective electrode wired to a processing unit (B), and an pH-sensitive electrode with a corresponding pH meter (C).


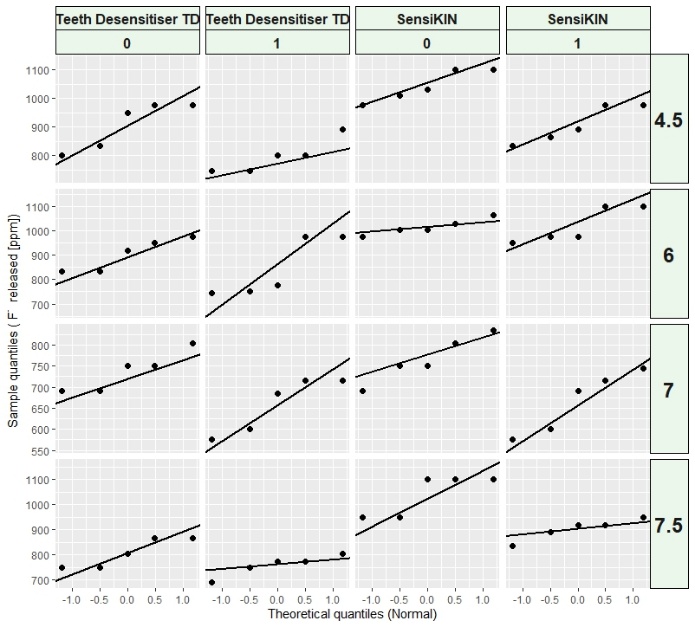

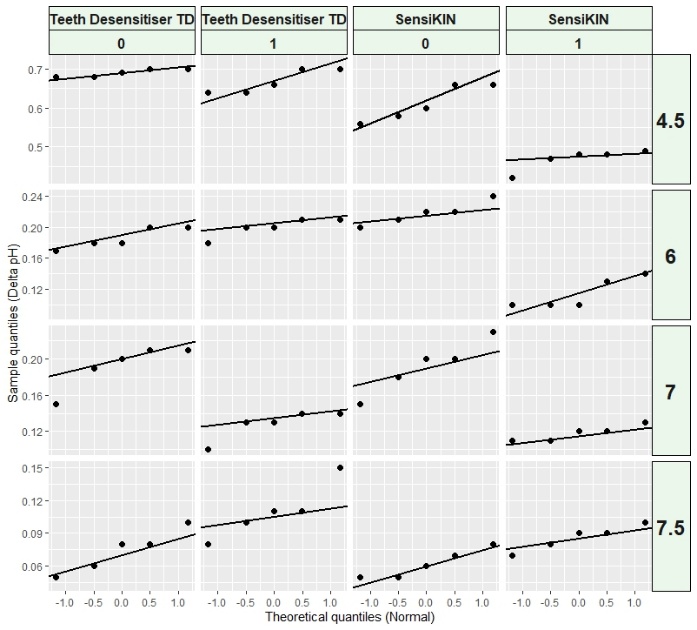


**Supplementary Figure 3.** Q-Q plots for acquired data without addition of TISAB (left: for F release, right: for pH change) pertaining to the validity of the ANOVA test.
